# Agentive linguistic framing affects responsibility assignments toward AIs and their creators

**DOI:** 10.3389/fpsyg.2025.1498958

**Published:** 2025-05-07

**Authors:** Dawson Petersen, Amit Almor

**Affiliations:** ^1^Linguistics Program, University of South Carolina, Columbia, SC, United States; ^2^Department of Psychology, University of South Carolina, Columbia, SC, United States

**Keywords:** linguistic framing, grammatical metaphor, agency, anthropomorphism, AI

## Abstract

Tech companies often use agentive language to describe their AIs (e.g., The Google Blog claims that, “Gemini can understand, explain and generate high-quality code,”). Psycholinguistic research has shown that violating animacy hierarchies by putting a nonhuman in this agentive subject position (i.e., grammatical metaphor) influences readers to perceive it as a causal agent. However, it is not yet known how this affects readers’ responsibility assignments toward AIs or the companies that make them. Furthermore, it is not known whether this effect relies on psychological anthropomorphism, or a more limited set of linguistic causal schemas. We investigated these questions by having participants read a short vignette in which “Dr. AI” gave dangerous health advice in one of two framing conditions (AI as Agent vs. AI as Instrument). Participants then rated how responsible the AI, the company, and the patients were for the outcome, and their own AI experience. We predicted that participants would assign more responsibility to the AI in the Agent condition, and that lower AI experience participants would assign higher responsibility to the AI because they would be more likely to anthropomorphize it. The results confirmed these predictions; we found an interaction between linguistic framing condition and AI experience such that lower AI experience participants assigned higher responsibility to the AI in the Agent condition than in the Instrument condition (*z* = 2.13, *p* = 0.032) while higher AI experience participants did not. Our findings suggest that the effects of agentive linguistic framing toward non-humans are decreased by domain experience because it decreases anthropomorphism.

## 1 Introduction

Linguists have long argued that the subject position of transitive clauses carries proto-agentive entailments – e.g., volition, sentience, causativity, etc. (Dowty, 1991). These entailments not only affect sentence processing, but also influence the situation models that hearers construct. For example, cross-linguistic differences in how frequently speakers of different languages use agentive language to describe accidents has been shown to predict how well participants remember the agent of accidental events (Fausey and Boroditsky, 2010; Fausey et al., 2011). The choice of grammatical subject, specifically, has been shown to affect readers’ perceptions of agency and responsibility. Unaccusative transitivity alternations (e.g., “the boy broke the window” vs. “the window broke”) allow speakers to choose whether or not to assign an agent for a specific event (Fausey and Boroditsky, 2011). Speakers are able to use these subtle syntactic alternations to manipulate the interpretive framework adopted by their hearers, without substantially altering propositional content (Thibodeau and Boroditsky, 2011; McGlynn and McGlone, 2019; Dragojevic et al., 2014). Psycholinguistic research has shown a linguistic framing effect in which the assignment of grammatical agency influences the situation models that readers construct in text interpretation. For example, Fausey and Boroditsky (2011) showed participants texts containing the sentences in (1) and (2).

(1) As Mrs. Smith reached to grab the napkin, she toppled the candle and ignited the whole tablecloth too!
(2) As Mrs. Smith reached to grab the napkin, the candle toppled and the whole tablecloth ignited too!

The sentences in (1) and (2) describe the same situation, but they differ in how they assign grammatical agency. In (1), Mrs. Smith is the agent of the transitive verb “topple.” In (2), after an unaccusative transition, the verb does not have an agent (though the reader can still infer that Mrs. Smith was the cause of the toppling). As predicted, Fausey and Boroditsky (2011) found that participants who read (1) assigned higher blame and financial liability to Mrs. Smith compared to participants who read (2). This finding has since been replicated by Tonković et al. (2022).

Notably, English allows speakers to violate the animacy hierarchy (Minkoff, 2000) by assigning this grammatical agency to inanimate entities in a phenomenon known as grammatical metaphor (Devrim, 2015). In these structures, an inanimate entity is not only the grammatical subject but also the agent of a transitive verb. For example, consider the sentences in (3) and (4).

(3) Doctors saved many lives by using Scan AI™ to identify early-stage cancer.
(4) Scan AI™ saved many lives by enabling doctors to identify early-stage cancer.

The sentences in (3) and (4) describe the same situation. However, they differ in how they assign grammatical agency. In (3), the agency for “saving lives” is assigned to the doctors while in (4) that agency is assigned to the inanimate AI. The use of grammatical metaphor has been shown to increase the responsibility assigned to radon gas (Dragojevic et al., 2014) and obesity (McGlynn and McGlone, 2019), to make educational materials about viruses more persuasive (McGlone et al., 2012), and to change how much autonomous behavior participants assign to unknown objects (Fausey and Boroditsky, 2007).

The effects of grammatical metaphor are uniquely interesting in the context of AI. During the deep-learning AI boom of the early 2020s, it became increasingly clear that people are highly prone to perceiving and interacting with AIs, and especially chatbots, as humanlike agents (i.e., anthropomorphizing them, Mitchell and Krakauer, 2023)—with some journalists and developers even going so far as to argue that LLMs are conscious agents (Tiku, 2022; Schwitzgebel and Shevlin, 2023). Of course, AI anthropomorphism is not new. Indeed, since Weizenbaum’s (1966) simple ELIZA chatbot, it has been known that people tend to assume that chatbots know more than they really do and are more capable than they really are (i.e., the ELIZA Effect, Hofstadter, 1995). However, the explosion of AI technology has seen AI developers frequently describe their models using agentive linguistic framing as in (5)–(7).

(5) ChatGPT sometimes writes plausible-sounding but incorrect or nonsensical answers (Open AI, 2022).
(6) Copilot promises to unlock productivity for everyone (Spataro, 2023).
(7) Gemini can understand, explain and generate high-quality code (Pichai and Hassabis, 2023).

AI anthropomorphism provides a unique opportunity to better understand the effects of grammatical metaphor. More specifically, while it has been shown that grammatical metaphor causes readers to derive proto-agentive entailments about non-humans, it is not clear whether it involves actually anthropomorphizing the target or if it relies on more limited linguistic causal schemas. The prevalence of AI anthropomorphism creates an opportunity answer this important theoretical question. If the effect of grammatical metaphor relies on anthropomorphism, we would expect factors that predict anthropomorphism (namely, domain experience and anthropomorphic disposition) to interact with a linguistic framing manipulation to produce a stronger effect.

Theories of anthropomorphism suggest that AI anthropomorphism may be a result of the difficulty people have building mechanistic mental models of AI (Epley et al., 2007; Dennett, 1987). According to Epley et al.’s (2007) three-factor theory and Dennett’s (1987) intentional stance approach, anthropomorphism can serve as a predictive strategy for interacting with unpredictable entities. In other words, treating an AI as an agent (with goals, beliefs, and means-end rationality) provides a framework for people to reason about an otherwise abstract system. Therefore, these accounts of anthropomorphism predict that experience with AI will moderate anthropomorphism because participants with more AI experience will find AI more predictable and so they will not need to rely on an agency framework to understand its behavior.

This further suggests that domain experience with AI could provide an “inoculating” effect against agentive linguistic framing. Because people with low AI experience do not have a robust mental model of how it works, they should be more likely to adopt the mental model suggested by the linguistic framing (i.e., adopting an anthropomorphic mental model when the AI is framed agentively and a mechanistic one when it is framed as a tool). In contrast, people with high AI experience already have a mental model of AI, and as a result, they should not only be less likely anthropomorphize to begin with, but they should also be less affected by the linguistic framing. If such an interaction were found, it would provide evidence that the effects of grammatical metaphor rely on the rich representations of agency and intention involved in anthropomorphism, not just linguistic causal schemas.

Recent work on the effects of linguistic framing on perceptions of robots provides preliminary evidence that such an inoculating effect exists. Kopp et al. (2022) found that factory workers perceived robots as more humanlike when they were described anthropomorphically (e.g., “[The robot] *Paul waits patiently* during employees’ lunch break”) as opposed to non-anthropomorphically (e.g., “[The robot] *UR-5* is *switched to idle mode* during employees’ lunch break”). However, Kopp et al. (2023) failed to replicate this effect with technology students. Kopp et al. (2023) suggest that this failure may be due to the students’ higher level of experience with robots; however, they note that the two studies utilized somewhat different experimental methods. As such, it is not clear whether domain experience modulates the effects of agentive linguistic framing as is predicted by theories of anthropomorphism.

Not only is there theoretical value to better understanding grammatical metaphor and its relationship to AI anthropomorphism, these questions also raise legal implications. Previous studies overwhelmingly focused on how grammatical metaphor affects participants’ perceptions technology, but they have not examined how grammatical metaphor affects participants’ perceptions of the technology’s creators. However, this is an issue of great practical importance. Some companies have already tried to argue that they are not legally responsible for information produced by their AIs (see Garcia, 2024), and if, as previous research suggests, linguistic framing can cause people to see AIs as responsible agents, they are likely to see the companies which create and deploy those AIs as less responsible.

In light of these issues, the current study investigates the interaction between agentive linguistic framing and domain experience with AI on participants’ responsibility assignments both to AIs and to the companies that create them. Specifically, we predict that (1) participants with lower AI experience will rate the AI as more responsible than participants with higher AI experience; (2) when the AI is framed as an agent, participants will rate it as more responsible and the company that made it as less responsible than when it is framed as a tool; and (3) participants with lower AI experience will be more affected by the linguistic framing manipulation than participants with higher AI experience.

## 2 Materials and methods

We tested these hypotheses using a judgment priming paradigm in which participants first read a short vignette in one of two linguistic framing conditions (Agent vs. Instrument) and then made judgments about it. The vignette (see Table 1) described how an AI language model “Dr. A.I.” gave dangerous health advice causing many patients to be hospitalized. The linguistic framing manipulation was achieved using grammatical metaphor (i.e., making the AI the grammatical subject of transitive clauses) as well as active/passive voice shifts. The two versions of the vignette were otherwise identical. After reading the vignette, participants were asked to rate on a scale from 1 to 100: (1) to what extent the AI, the company that created it, and the patients were each responsible for the outcome, and (2) how much experience they had with AI. Finally, participants completed the Individual Differences in Anthropomorphism Questionnaire (IDAQ) (Waytz et al., 2010), and then were asked to retell the story from the vignette in as much detail as they could remember. This recall data was used to ensure that participants read and understood the vignette in sufficient detail. The full survey is available through the OSF repository.

**Table 1:** The agent and instrument condition vignettes.

Agent condition	Instrument condition
In 2023, an A.I. language model called "Dr. A.I." captured widespread attention after being released by a tech company called Health A.I. Dr. A.I. tried to provide accurate, tailored medical advice based on what it knew about users' symptoms and medical histories. However, in 2024, Dr. A.I. made an error when it recommended a dangerous home cure for a common cold. Several people who followed this advice were hospitalized, and one person died. The families of the people who were hospitalized are preparing a large lawsuit against Health A.I.	In 2023, a tech company called Health A.I. captured widespread attention after they created an A.I. language model called "Dr. A.I." Dr. A.I. was designed to provide accurate, tailored medical advice based on the company's data about users' symptoms and medical histories. However, in 2024, a recommendation for a dangerous home cure for a common cold was generated by Dr A.I. Several people who followed this advice were hospitalized, and one person died. The families of the people who were hospitalized are preparing a large lawsuit against Health A.I.

Key differences between the two versions are underlined here for clarity, but were not visible to participants.

We recruited 157 participants from psychology and linguistics classes at the University of South Carolina. Of these, 35 were excluded for failure to complete the study or failure to recall the key details of the vignette, resulting in a final sample size of 122. Participants were considered to not recall key details if they wrote that they did not remember, or if they grossly misremembered the main characters and events of the story—especially if it was not clear that they realized an AI was involved (e.g., “An online Dr. passed out a medication,” “The company failed in the aspect of a home invasion”). Participants who merely mixed up the names of the company and the AI were not excluded.

## 3 Results

The data were analyzed in R 4.3.0 (R Core Team, 2023). Participants generally rated themselves as having low AI experience, with self-rated experience falling into a heavily right-skewed distribution (*M* = 19.8, *SD* = 24.6, *Median* = 10, *Mode* = 0). As expected, experience was negatively correlated with AI responsibility assignments (*R* = −0.29, *p* = 0.001), but had no correlation with company (*R* = −0.03, *p* = 0.728) or patient responsibility assignments (*R* = 0.02, *p* = 0.819). Overall, participants assigned the most responsibility to the company (*M* = 70, *SD* = 23), followed by the AI (*M* = 49, *SD* = 35), and the least to the patients (*M* = 43, *SD* = 26). However, responsibility assignments toward all three targets were non-normally distributed. Responsibility assignments toward the company showed a strong leftward skew with a primary mode of 100 (*frequency* = 18) and a secondary mode of 50 (*frequency* = 16). Responsibility assignments toward the AI were trimodal with a primary mode of 0 (*frequency* = 16), and secondary modes of 50 (*frequency* = 12) and 100 (*frequency* = 11). Responsibility assignments toward the patients were trimodally distributed and had an overall rightward skew, with a primary mode of 50 (*frequency* = 12) and secondary modes of 70 (*frequency* = 10) and 10 (*frequency* = 9). Additional descriptive statistics are available in Table 2.

**Table 2:** The mean, median, and standard deviations of participants’ responsibility ratings toward the company, the AI, and the patients, by condition and quartile of AI experience.

Group	n	Mean	Median	Mode(s) [*frequency*]	SD
Company responsibility ratings					
Agent condition	57	72.07	72	100[*f* = 8]	22.04
Instrument condition	65	69.29	71	50[*f* = 12] 100[*f* = 10]	23.38
First quartile of AI experience	31	70.06	71	100[*f* = 5]	22.38
Second quartile of AI experience	31	69.67	70	50[*f* = 5]	22.80
Third quartile of AI experience	30	76.77	80.5	100[*f* = 7]	21.62
Fourth quartile of AI experience	30	65.9	70	50[*f* = 4]	23.80
Overall	122	70.59	71	100[*f* = 18] 50[*f* = 16]	22.82
AI responsibility ratings					
Agent condition	57	49.89	50	0[*f* =10]	37.01
Instrument condition	65	49.14	50	0[*f* =10] 50[*f* =10]	32.66
First quartile of AI experience	31	59.64	70	100[*f* =5] 80[*f* =4] 0[*f* =4]	34.44
Second quartile of AI experience	31	54.06	50	50[*f* =5]	32.54
Third quartile of AI experience	30	49.93	52.5	0[*f* = 6]	37.56
Fourth quartile of AI experience	30	33.83	36.5	0[*f* =7]	29.67
Overall	122	49.49	50	0[*f* =16] 50[*f* =12] 100[*f* = 11]	34.62
Patient responsibility ratings					
Agent condition	57	42.82	41	70[*f* = 5]	27.18
Instrument condition	65	42.09	47	50[*f* = 10]	24.79
First quartile of AI experience	31	45.19	50	0[*f* = 4] 50[*f* = 4]	25.50
Second quartile of AI experience	31	33.64	30	5[*f* = 4]	27.51
Third quartile of AI experience	30	43.77	44	10[*f* = 4] 70[*f* = 4]	26.24
Fourth quartile of AI experience	30	47.33	48.5	20[*f* = 3] 30[*f* = 3] 50[*f* = 3]	22.83
Overall	122	42.43	42.5	50[*f* = 12] 70[*f* = 10] 10[*f* = 9]	25.82

**Figure 1:**
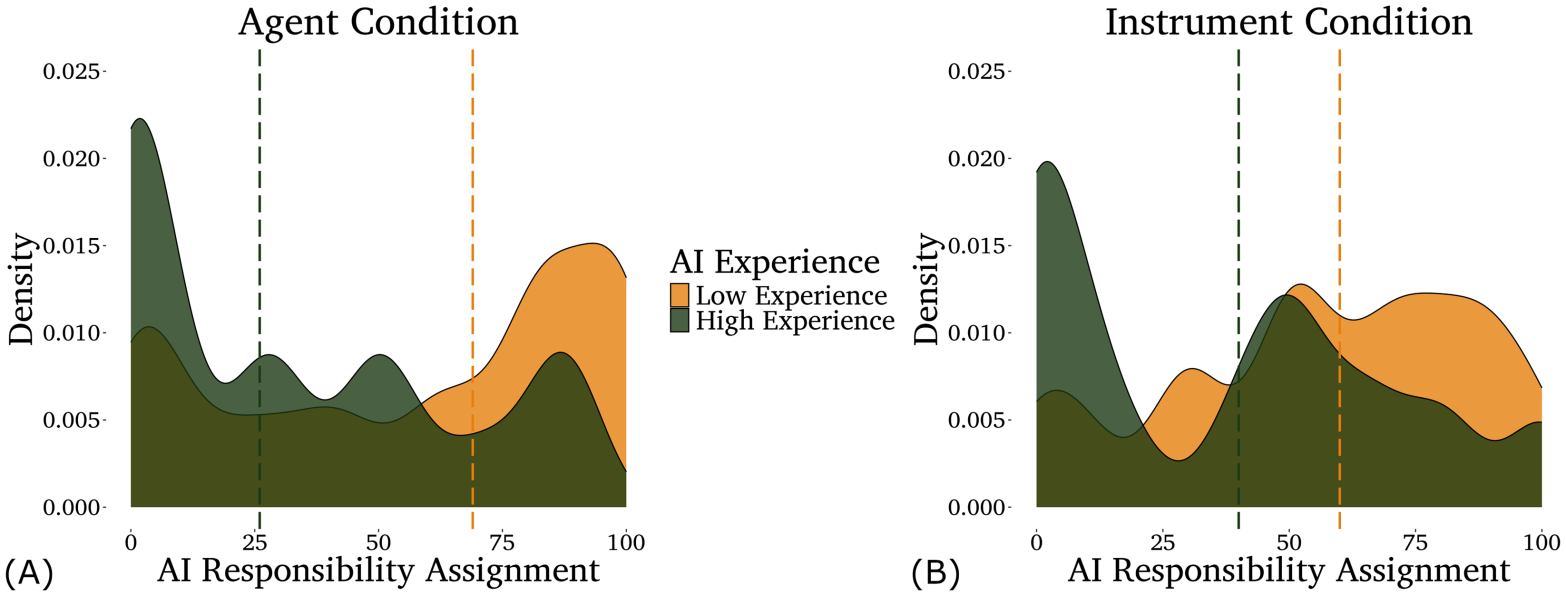
Density plots of the responsibility assigned to the AI in the agent **(A)** and instrument **(B)** conditions by participants with different levels of self-rated AI experience—low in light/gold (below the mean, *n* = 80) and high in dark/green (above the mean, *n* = 42). Note that AI experience was analyzed as a continuous variable, although it is displayed as a categorical variable here for the purpose of data visualization. Medians for each group are shown by the dashed lines.

**Figure 2:**
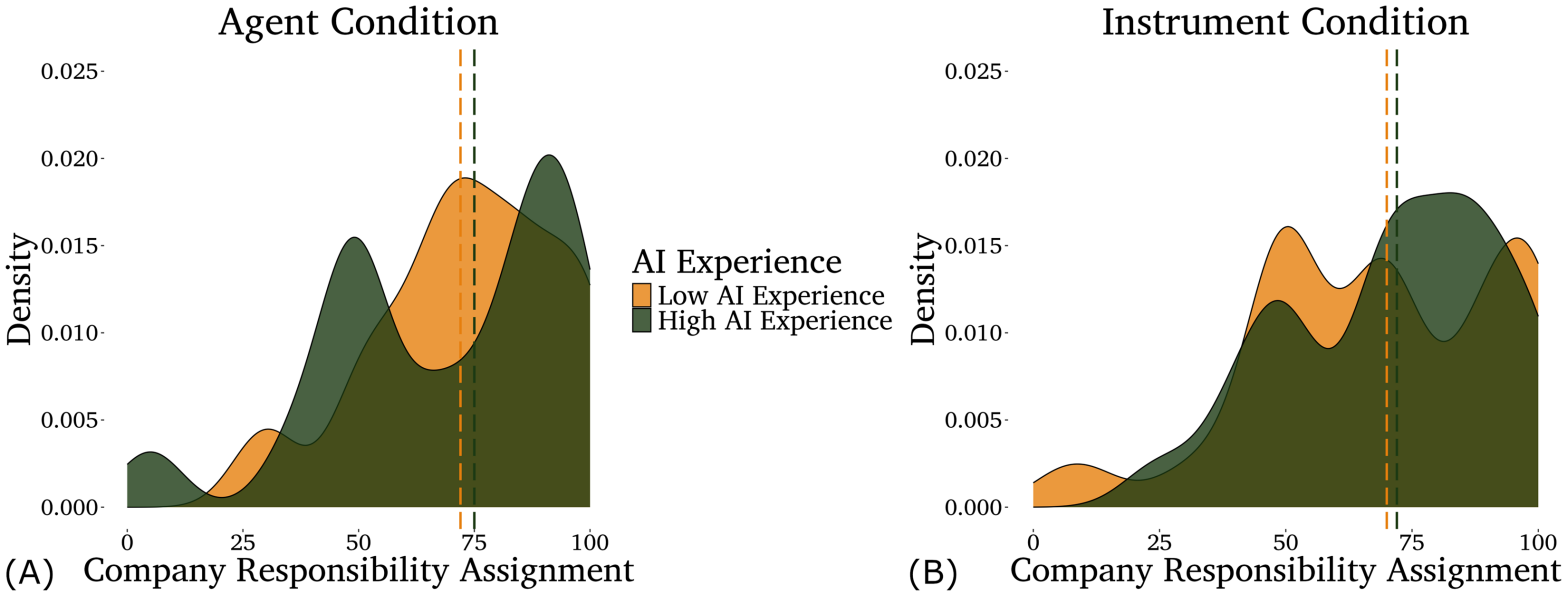
Density plots of the responsibility assigned to the company in the agent **(A)** and instrument **(B)** conditions by participants with different levels of self-rated AI experience—low in light/gold (below the mean, *n* = 80) and high in dark/green (above the mean, *n* = 42). Note that AI experience was analyzed as a continuous variable, although it is displayed as a categorical variable here for the purpose of data visualization. Medians for each group are shown by the dashed lines.

**Figure 3:**
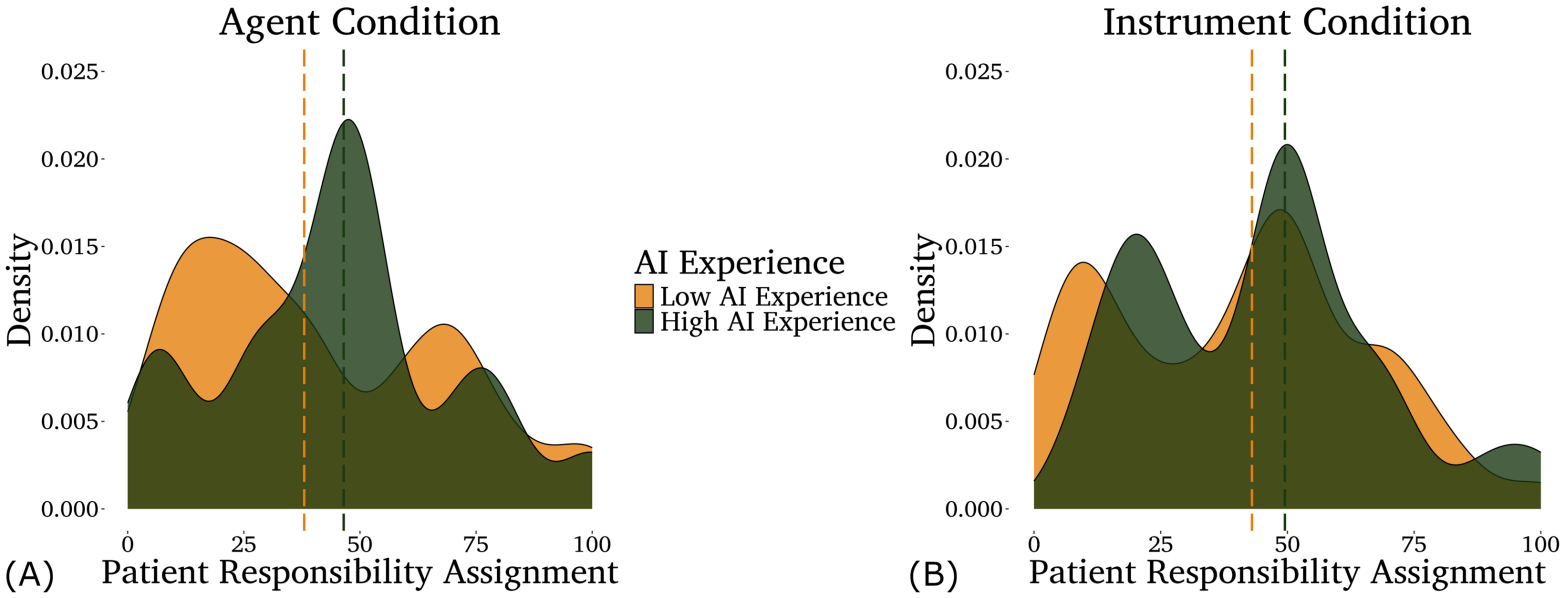
Density plots of the responsibility assigned to the patients in the agent **(A)** and instrument **(B)** conditions by participants with different levels of self-rated AI experience—low in light/gold (below the mean, *n* = 80) and high in dark/green (above the mean, *n* = 42). Note that AI experience was analyzed as a continuous variable, although it is displayed as a categorical variable here for the purpose of data visualization. Medians for each group are shown by the dashed lines.

Note that these measures of central tendency are not highly informative due to the non-normal distribution of the data (see Figures 1–3).

### 3.1 Main analysis

Mean differences between the two framing conditions were not observed in responsibility ratings (see Table 2). However, these measures of central tendency are not highly informative because of the non-normal distributions of the rating data. Furthermore, standard operations such taking the natural log were not able to sufficiently normalize the responsibility rating data for parametric analysis. Therefore, we analyzed the responsibility rating data using cumulative link regression models (Agresti, 2012) implemented with the *ordinal* package 4.1 (Christensen, 2022) in R. The responsibility assigned to the AI, the company, and the patients were each modeled separately as dependent variables. For each dependent variable, condition (Agent vs. Instrument), log self-rated AI experience, and participants’ IDAQ scores were considered as predictors. Alternative models were compared using Akaike’s information criterion (AIC) (Akaike, 2011), and optimal models were selected.

For AI responsibility, the optimal model included only the interaction between condition and AI experience. IDAQ scores failed to improve model fit (*p* = 0.169). We found a main effect of AI experience (*z* = −3.68, *p* < 0.001) such that participants with less AI experience assigned more responsibility to the AI and an interaction between framing condition and AI experience (*z* = 2.13, *p* = 0.032) such that low AI experience participants assigned more responsibility to the AI in the Agent condition than the Instrument condition, while high AI experience participants did not (illustrated in Figure 1). The main effect of framing condition was only marginally significant (*z* = −1.86, *p* = 0.06).

For company responsibility, once again the optimal model included only the interaction between condition and AI experience, and IDAQ scores failed to improve model fit (*p* = 0.559). We found a main effect of condition (*z* = −2.01, *p* = 0.036) such that participants in the Agent condition assigned less responsibility to the company than participants in the Instrument condition, and an interaction between condition and AI experience (*z* = 2.42, *p* = 0.015) such that the main effect of condition was stronger for participants with high AI experience (illustrated in Figure 2).

For patient responsibility, the optimal model included only AI experience. However, we found no significant effect of AI experience in that model. Indeed, even using maximal models of patient responsibility assignments, we did not find any significant main effects or interactions between our predictors (illustrated in Figure 3).

### 3.2 Effect size stabilization analysis

Because we did not have an *a priori* expectation of effect size, we were not able to perform an *a priori* power analysis to select an appropriate sample size. Therefore, in order to determine whether or not our experiment was sufficiently powered to detect real effects in the population, we performed an effect size stabilization analysis, following the approach endorsed by Anderson et al. (2022). Anderson et al. showed that if one continuously calculates model effect size as participants are added to the sample, data collection can be safely ended when the effect size stabilizes without the introduction of statistical bias (i.e., p-hacking). In other words, when the effect size stabilizes, it likely represents the true effect size in the population.

**Figure 4:**
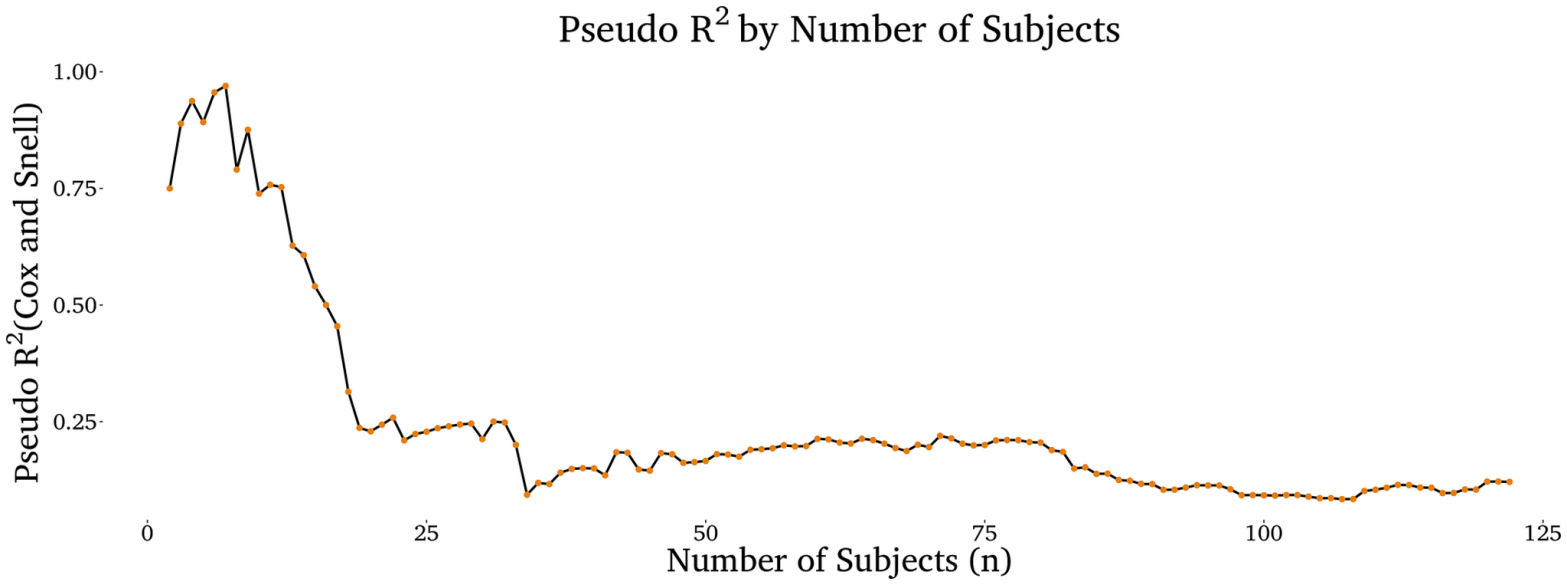
Pseudo R^2^ measure of effect size (Cox and Snell) by the number of subjects included in the analysis of the responsibility assigned to the AI. The results show that Cox and Snell stabilized at *R^2^* = ~0.12 when data collection was stopped at *n* = 122.

Because the ordinal cumulative link models which we used output log likelihoods, the most natural measures of effect size are Pseudo R^2^ measures such as Cox and Snell (Cox and Snell, 2018). Therefore, we reperformed our analysis of the responsibility assignments to the AI, and then we computed Cox and Snell each time a new datapoint was added to the sample using the *rcompanion* package 2.5.0 (Mangiafico, 2017). The results (see Figure 4) show that the effect size had changed minimally (less than +/− 0.02) for fifteen consecutive participants when we stopped data collection at n = 122. Therefore, we conclude, following Anderson et al. (2022), that this sample size was sufficient to detect the approximate true effect size in the population.

## 4 Discussion

Overall, our findings are generally consistent with our hypotheses. Firstly, we found that, as predicted by theories of anthropomorphism such as Epley et al. (2007) and Dennett (1987), participants with lower AI experience assigned higher responsibility to the AI than participants with higher AI experience. Secondly, we found a linguistic framing effect such that assigning grammatical agency to the AI resulted in higher responsibility assignments to the AI and lower responsibility assignments to the company that created it. Crucially, these linguistic framing effects were dependent on AI experience. Specifically, we found that only lower AI experience participants assigned higher responsibility to the AI in the Agent condition compared to the Instrument condition, while higher AI experience participants showed the opposite trend. This finding provides evidence that domain experience can indeed have an inoculating effect against the linguistic framing effects of grammatical metaphor. In turn, this provides evidence that the effects of grammatical metaphor involve anthropomorphism, as domain experience would not be expected to modulate the effects of grammatical metaphor if they occurred only at the level of linguistic causal schemas. Instead, this finding provides additional evidence that the effects of grammatical metaphor occur in more general processes of situation model construction, as has been argued by Fausey and Boroditsky (2011) to be the case for other types of agentive linguistic framing.

Interestingly, however, we did not find any effects of the IDAQ (Waytz et al., 2010) on AI responsibility assignments. This finding runs contrary to the predictions of Epley et al.’s (2007) three-factor theory; however, it replicates the findings of previous studies (e.g., Tahiroglu and Taylor, 2019; Hortensius et al., 2021) which found that the IDAQ is a poor predictor of participants’ tendency to make anthropomorphic attributions about specific situations. Taken together, these findings suggest that more generalized anthropomorphic beliefs (as measured by the IDAQ) may not strongly influence more particularized anthropomorphic attributions (e.g., contextual, causal explanations of behavior, Hilton, 2007). This finding is further congruent with Thellman and Ziemke (2019) position that anthropomorphic attributions are ontologically non-committal. In other words, people can adopt anthropomorphic causal explanations of an inanimate entity’s behavior, even when these explanations contradict their explicit beliefs about its abilities.

Additionally, we found that all participants assigned less responsibility to the company in the AI as Agent condition than in the AI as Instrument condition, regardless of experience level. This stands in contrast to our finding that AI experience decreased the effects of agentive linguistic framing on AI responsibility assignments. Not only was this not the case for company responsibility assignments, we even found that the framing effect was stronger for higher experience participants than for lower experience ones. This finding has both theoretical and practical implications. Firstly, it helps us to better understand the involvement of anthropomorphism in the effects of grammatical metaphor. It shows that the higher experience participants did form different causal situation models as a result of the grammatical metaphor, and therefore, suggests that they inhibited their responsibility assignments to the AI in the Agent condition because they were unwilling to anthropomorphize it. If this is the case, it complicates the picture somewhat regarding the inoculating effect of experience that we have proposed—as AI experience appears to only inoculate against one of the effects of the grammatical metaphor (higher responsibility to the AI) and not the other (lower responsibility to the creator). The practical upshot of this is that people with high AI experience may still be easily manipulated by grammatical metaphor to perceive tech companies as less responsible for their AIs’ behavior.

One important limitation of this study, however, is the range of experience levels included in our sample. Our sample was composed primarily of first- and second-year undergraduate students with generally low self-rated AI experience (*M* = 19.8, *SD* = 24.6, *Median* = 10, *Mode* = 0). Although we found interesting differences between higher and lower experience participants within this range, further work is required to understand how these effects appear in truly high experience participants within the general population (e.g., tech workers, computer scientists, etc.). If the relationship between linguistic framing and AI experience is in someway nonlinear, we may find that such individuals behave quite differently from the higher experience participants within our sample.

Our results further raise questions regarding how specific properties of AI systems (and interaction with them) affects participants’ agency assignments. One particularly interesting factor is language use. Weizenbaum’s (1966) ELIZA effect suggests that AI language use has powerful effects on users’ perceptions of its agency. If this is indeed the case, we may find that participants respond differently to chatbots (e.g., “Dr. A.I.”) compared to AIs used for image recognition/generation, driving, financial analysis etc.—despite the similarities in the underlying technology. Such research should further seek to understand how interaction with language AIs affects users’ perceptions. For example, restricting a chatbot’s ability to use first-person pronouns (“I,” “my,” etc.) may significantly decrease users’ perception of its agency.

Finally, our findings have important practical and ethical implications for how we talk about AI. They show that linguistically framing AIs as agents influences lower experience people to anthropomorphize the AIs and influences all people to consider the companies which create them less responsible for their mistakes. Historically, authors disagree as to the extent to which such anthropomorphism of AI is desirable (Deshpande et al., 2023) or dangerous (Hasan, 2023), and indeed, some AI researchers even advocate including anthropomorphic features to increase user trust in the AI (Song and Luximon, 2020). Given our findings, we argue that encouraging the anthropomorphism of AI by using agentive linguistic framing is dangerous as it can cause even experienced individuals to fail to hold AI companies accountable when their creations cause harm.

## Data Availability

The datasets presented in this study can be found in online repositories. The names of the repository/repositories and accession number(s) can be found at: https://doi.org/10.17605/OSF.IO/7MGEV.
